# Five new records of bee flies (Bombyliidae, Diptera) from Saudi Arabia with zoogeographical remarks

**DOI:** 10.3897/zookeys.489.8794

**Published:** 2015-03-23

**Authors:** Magdi S. El-Hawagry, Hathal M. Al Dhafer

**Affiliations:** 1 Entomology Department, Faculty of Science, Cairo University, Egypt; 2 King Saud University, College of Food and Agricultural Sciences, Riyadh, Kingdom of Saudi Arabia

**Keywords:** Asir, Abha, Garf Raydah Protected Area, Baha, Jabal Shada Al A’Ala Protected Area, Tihama, Afrotropical

## Abstract

Five bee-fly species (Bombyliidae, Diptera) have been listed in this paper as new to the Kingdom of Saudi Arabia. Four of the recorded species have been identified to the level of species, namely: *Bombomyia discoidea* (Fabricius, 1794), *Spogostylum candidum* (Sack, 1909), *Exoprosopa linearis* Bezzi, 1924, and *Exoprosopa minos* (Meigen, 1804), while the fifth one only to genus, *Desmatoneura* sp. The species have been collected from Al-Baha and Asir Provinces in the south-western part of the Kingdom. One of the four identified species, *Exoprosopa linearis*, has an Afrotropical affinity, and another two, *Spogostylum candidum* and *Bombomyia discoidea*, have considerable Afrotropical distributions, and this result agrees to some extent with studies considering these parts of the Arabian Peninsula, including Al-Baha and Asir Provinces, having Afrotropical influences and may be included in the Afrotropical Region rather than in the Palaearctic Region or the Eremic zone.

## Introduction

**Figure 1.**
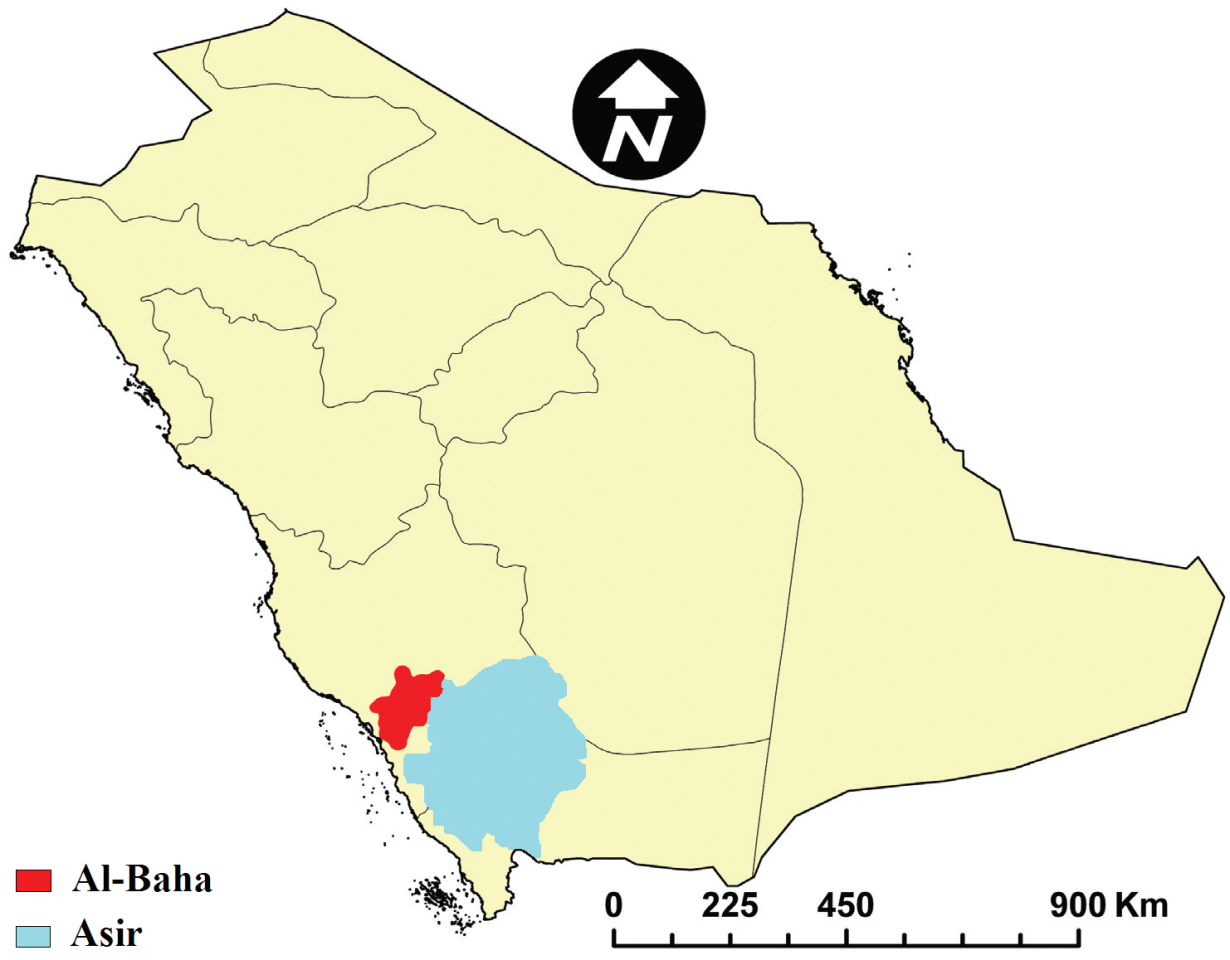
Map of Saudi Arabia showing Al-Baha and Asir Provinces.

Al-Baha and Asir are two neighboring provinces (Fig. 1) situated in the south-western part of the Kingdom of Saudi Arabia consisting together about 91362 km^2^, and characterized by natural tree cover and agricultural plateaus. The two provinces are similarly divided into two main sectors, a lowland at the west which forms part of the coastal plain extending from north to south, known as “Tihama”, and a mountainous area with an elevation of 1500 to about 3000 m above sea level at the east, known as “Al-Sarat” or “Al-Sarah” which forms part of the Al-Sarawat Mountains range (Alahmed et al. 2010, Ibrahim and Abdoon 2005, and El-Hawagry et al. 2013).

The climate in Al Baha Province is generally moderate in summer and cold in winter with average monthly temperatures ranging between 12–23 °C. While in Asir Province, the climate is moderate with average monthly temperatures ranging between 7–30 °C. In the lowland coastal plain, Tihama, the climate is hot in summer, warm in spring and mild in winter, with relative humidity (RH) ranging between 52–67% in Al-Baha Province and up to 90% in Asir Province, and a rainfall less than 100 mm annually in both. While in the mountainous area, Al-Sarah, the weather is generally cooler due to its high altitude, in addition to the formation of clouds and fog accompanied by thunderstorms in winter. The rainfall is throughout the year in the mountainous area (Al-Sarah) with an annual average of 405 mm in Al-Baha Province and 342 mm in Asir Province (Ibrahim and Abdoon 2005; Omer 1996 and websites: http://www.tititudorancea.com/z/weather_al_baha_saudi_arabia.htm).

Many authors include parts of the Arabian Peninsula in the Afrotropical Region, but there is no agreement as to how much. Crosskey (1980) used the northern boundaries of Yemen as the regional boundary between the Afrotropical and Palaearctic parts in the Arabian Peninsula. Sclater (1858) and Wallace (1876) proposed the classical zoogeographical regions and placed the northern border of the Afrotropical Region along the Tropic of Cancer; thus, Al-Baha and Asir Provinces were included in the Afrotropical Region (Hölzel 1998). However, according to Uvarov (1938), Greathead (1980, 1988), and Larsen (1984) this area should be united with the central Arabian deserts which are either considered as a part of the Palaearctic, or as an autonomous Eremic or Eremian zone (also called the Saharo-Sindian faunal region). Recently, extensive sampling of insects in the Arabian Peninsula by many authors, especially in Yemen, Oman, the United Arab Emirates and south-western mountains of Saudi Arabia, indicated that Sclater’s (1858) and Wallace’s (1876) concept of the extent of the Afrotropical Arabian Peninsula is more accurate than Crosskey’s (1980) limited concept of Yemen alone (Kirk-Spriggs and McGregor 2009). All these facts undoubtedly reflected somehow on the insect faunal composition in Al-Baha and Asir Provinces (El-Hawagry et al. 2013).

Greathead (1980 & 1988) recorded 100 bee-fly species and subspecies in Saudi Arabia out of 149 in the entire Arabian Peninsula, in addition to 4 species subsequently recorded by El-Hawagry et al. (2013) and another one was recently described by El-Hawagry and Al Dhafer (2014). Through our collecting trips for the present study, we have collected 15 bee-fly species from Al-Baha Province and 12 species from Asir Province. Five of the collected species are treated in the present study as new to the Kingdom of Saudi Arabia.

## Material and methods

Material of the present study has been collected occasionally from different localities in Al-Baha Province (Al-Mekhwa, Aqabet Al Baha-Tihama, Ghabet Shahba, Jabal Shada Al A’Ala Protected Area) and Asir Province (Garf Raydah Protected Area) in 2013 and 2014 by the authors using aerial nets. All sites of collection were generally rich in acacia, cactus, olive, juniper and alder buckthorn trees, and support an exceptionally rich flora, with approximately 500 plant species recorded, including 63 key plant taxa including endemics and Afrotropical relicts.

The global distributions of species were matched to that provided by Evenhuis and Greathead (1999). Efflatoun (1945), Greathead and Evenhuis (2001), and El-Hawagry et al. (2000) have been consulted to identify the genera and species.

### Abbreviations of museums

EFC Efflatoun collection, Entomology Department, Faculty of Science, Cairo University, Egypt.

KSMA King Saud University Museum of Arthropod Collection, Riyadh, Saudi Arabia.

## Results

Five bee-fly species are listed, which have not been recorded from Saudi Arabia before. In addition to these newly recorded taxa, 15 species from Al-Baha and 12 species from Asir Province were collected that have been previously recorded in Saudi Arabia (see El-Hawagry et al. 2013; El-Hawagry and Al Dhafer 2014 and Greathead 1980 & 1988). Four of the newly recorded species are identified to the species level, but the 5th could not be determined to that level. One of the four identified species, *Exoprosopa linearis* Bezzi, 1924, has an Afrotropical affinity, and another two, *Spogostylum candidum* (Sack, 1909) and *Bombomyia discoidea* (Fabricius, 1794), have considerable Afrotropical distributions. This result agrees to some extent with studies considering that parts of the Arabian Peninsula, including Al-Baha and Asir Provinces have Afrotropical influences and may be included in the Afrotropical Region rather than in the Palaearctic Region or the Eremic zone, and the northern limit of the Afrotropical Region should be placed along the Tropic of Cancer, about 200 km north to Al-Baha (El-Hawagry et al. 2013; Hölzel 1998; Sclater 1858; Wallace 1876).

### List of newly recorded species

#### Family Bombyliidae

##### Subfamily Bombyliinae

###### Tribe Bombyliini

####### Bombomyia discoidea

Taxon classificationAnimaliaDipteraBombyliidae

(Fabricius, 1794)

Figures 2–6

######## Remarks.

This is a robust species over 10 mm in length; with body, legs, and all spines and spicules black; with uniformly long hair on abdomen black at base, white at apex; thorax of female with gray to orange-brown hairs.

######## Distribution.

**Afrotropical:** Botswana, Burundi, Chad, Congo, Eritrea, Ethiopia, Gambia, Ghana, Kenya, Malawi, Mali, Mozambique, Namibia, Niger, Nigeria, Oman, Saudi Arabia (South-western part), Senegal, South Africa, Swaziland, Tanzania, Togo, Uganda, Yemen, Zambia, Zimbabwe. **Palaearctic:** Algeria, Armenia, Austria, Azerbaijan, China, France, Greece, Hungary, Iran, Israel, Italy, Mongolia, Russia, Spain, Turkey, Turkmenistan, Ukraine, Uzbekistan.

######## Material examined.

1 female, Al-Baha Province, Ghabet Shahba [20.02.723N,41.28.565E, 2324m], 20.V.2013, (El-Hawagry); 1 female, same data, 2.VI.2013; 1 female, Asir Province, Garf Raydah Protected Area [18°11.884'N, 42°24.435'E, 2387 m], 6.VI.2014, (El-Hawagry). All deposited in EFC.

**Figure 2–6.**
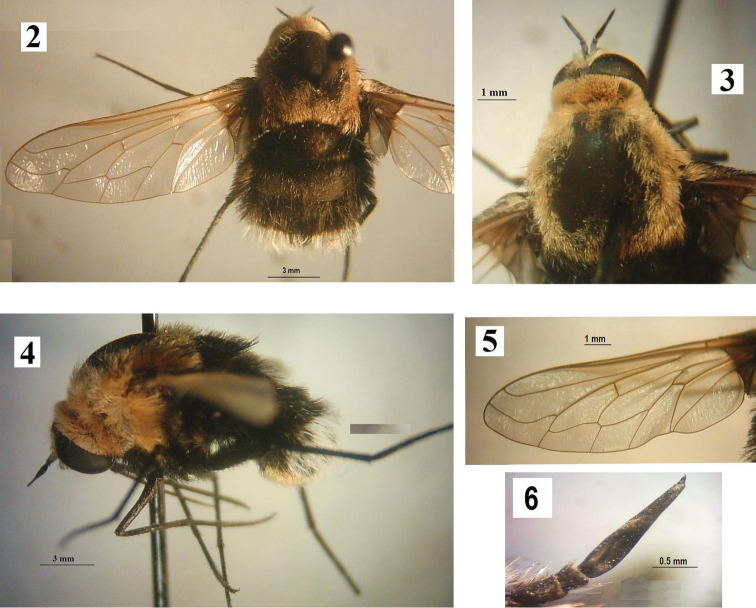
*Bombomyia discoidea*, Female: **2** Dorsal view **3** Head and thorax **4** Lateral view **5** Wing **6** Antenna.

##### Subfamily Anthracinae

###### Tribe Anthracini

####### Spogostylum candidum

Taxon classificationAnimaliaDipteraBombyliidae

Sack, 1909

Figures 7–8

######## Remarks.

The individuals of this species exhibit considerable variations in size, usually more than 10 mm in length. It can be distinguished from other species of the genus by the absence of alternating tufts of hairs on sides of abdomen; some long black hairs usually found on sides of 2^nd^ tergite but not in form of tufts; last three tergites extensively covered with dense white scales; lower part of face, above peristomal ridge, with long yellowish white hairs only; and aedeagus longer than aedeagal sheath.

######## Distribution.

**Afrotropical:** Egypt [as ”Gebel Elba”], Saudi Arabia (South-western part), United Arab Emirates. **Oriental:** Pakistan. **Palaearctic:** Iran, Turkey.

######## Material examined.

2 males, Al-Baha Province, Jabal Shada Al A’Ala Protected Area [19°50.710'N, 41°18.267'E, 1474 m], 4.VI.2014, (El-Hawagry). Deposited in KSMA.

**Figures 7–11.**
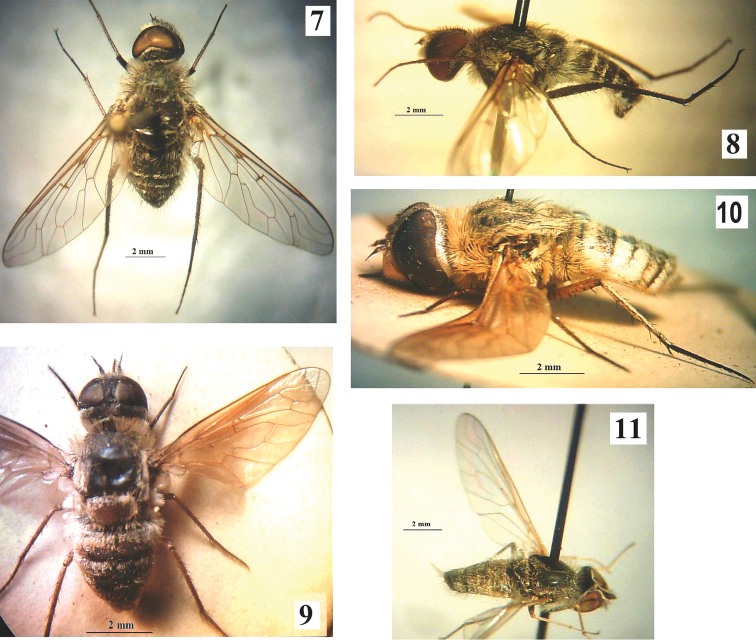
**7** *Spogostylum candidum*, male, dorsal view **8** Same, lateral view **9** *Exoprosopa minos*, female, dorsal view **10** Same, lateral view **11** *Desmatoneura* sp., male, dorso-lateral view.

###### Tribe Exoprosopini

####### Exoprosopa linearis

Taxon classificationAnimaliaDipteraBombyliidae

Bezzi, 1924

######## Remarks.

A single female in a poor condition has been collected. This species is easily distinguished by the wholly brownish infuscated wing, which tends to be darker at fore border and along veins; also by the abdomen which is narrow parallel sided with contrasting bands of black and white scales.

######## Distribution.

**Afrotropical:** Eritrea, Oman, Saudi Arabia (South-western part), Yemen.

######## Material examined.

1 female, Al-Baha Province, Al-Mekhwa [19.81328°N, 41.44073°E, 455m], 27.III.2013, (El-Hawagry). Deposited in EFC.

####### Exoprosopa minos

Taxon classificationAnimaliaDipteraBombyliidae

(Meigen, 1804)

Figures 9–10

######## Remarks.

This species is distinguished by the remarkable transverse bands of white scales on the abdominal tergites, by the brownish infuscation at the fore border and base of wing, and by the black antennae and legs.

Considering the south-western part of Saudi Arabia as included in the Afrotropical Region, this is the first record of this species from the Afrotropical Region.

######## Distribution.

**Afrotropical:** Saudi Arabia (South-westrern part). **Palaearctic:** Algeria, Armenia, Austria, Azerbaijan, Croatia, Czech Republic, Egypt, France, Georgia, Germany, Greece, Hungary, Iran, Israel, Palestine (West Bank), Italy, Kazakhstan, Kyrgyzstan, Lebanon, Libya, Moldova, Morocco, Poland, Romania, Russia, Slovakia, Spain, Syria, Tajikistan, Tunisia, Turkey, Turkmenistan, Ukraine, Uzbekistan.

######## Material examined.

1 male and 2 females, Al-Baha Province, Aqabet Al Baha-Tihama [20.00000°N, 41.43758°E, 1300 m], VI-V.2013, (El-Hawagry). Deposited in EFC.

###### Tribe Xeramoebini

####### Desmatoneura sp.

Taxon classificationAnimaliaDipteraBombyliidae

Figure 11

######## Remarks.

A single male agreeing with characters of genus *Desmatoneura* Williston, 1895 has been collected. Greathead (1980 & 1988) questionably recorded *Desmatoneura frontalis* (Wiedemann, 1828) from Oman; *Desmatoneura brevipennis* (Bezzi, 1924) from Yemen, Oman, and United Arab Emirates; and an unidentified species from United Arab Emirates. Evenhuis and Greathead (1999) recorded *Desmatoneura frontalis* (Wiedemann, 1828) from Saudi Arabia. Species in this genus are little-known and the present one is probably new but more specimens are required to ensure that.

######## Material examined.

1 male, Al-Baha Province, Jabal Shada Al A’Ala Protected Area [19°50.710'N, 41°18.267'E, 1474 m], 4.VI.2014, (El-Hawagry). Deposited in KSMA.

## Supplementary Material

XML Treatment for Bombomyia discoidea

XML Treatment for Spogostylum candidum

XML Treatment for Exoprosopa linearis

XML Treatment for Exoprosopa minos

XML Treatment for Desmatoneura sp.
